# Treatment of hepatitis C virus infection in the future

**DOI:** 10.1186/2001-1326-2-9

**Published:** 2013-04-11

**Authors:** Tatsuo Kanda, Osamu Yokosuka, Masao Omata

**Affiliations:** 1 Department of Gastroenterology and Nephrology, Chiba University, Graduate School of Medicine, 1-8-1 Inohana, Chuo-ku, Chiba (260-8670), Japan; 2 Yamanashi Hospitals (Central and Kita) Organization, 1-1-1 Fujimi, Kofu-shi, Yamanashi (400-8506), Japan; 3 University of Tokyo, 7-3-1, Hongo, Bunkyo-ku, Tokyo (113-8655), Japan

**Keywords:** HCV, Telaprevir, Boceprevir, Sofosbuvir, Daclatasvir

## Abstract

Two direct-acting antivirals (DAAs) against hepatitis C virus (HCV): telaprevir and boceprevir, are now available in combination with peginterferon plus ribavirin for the treatment of chronic hepatitis C infection. Although these drugs are potent inhibitors of HCV replication, they occasionally result in severe adverse events. In the present clinical trials, in their stead, several second-generation DAAs are being investigated. Most of them are being viewed with high expectations, but they also require the combination with peginterferon plus ribavirin. In the near future, we might be using all-oral DAAs and interferon-free regimens for the treatment of HCV-infected patients, and these would be potent inhibitors of HCV and have less adverse events.

## Review

### Introduction

Hepatitis C virus (HCV) chronically infects an estimated 170 million people worldwide [1]. HCV infection is one of the major causes of end-stage liver disease and hepatocellular carcinoma (HCC) worldwide [2-4]. Approximately 30% of patients who develop acute hepatitis C recover spontaneously, signaled by improved symptoms, normalized liver-related chemistries, loss of HCV RNA from serum, and the development of HCV antibody [5-7]. In chronic hepatitis C, the progression of liver fibrosis is slow, but steady. It has been reported that the progression rate of liver fibrosis is 0.10-0.13 U/year in untreated patients [8]. Progression of chronic HCV infection is not linear in time, probably because many cofactors are involved in changing the rate of development of fibrosis, cirrhosis, and HCC [6]. Cirrhosis rates become significant after 20 years of HCV infection. About 20-30% of patients could develop a progressive liver disease leading to cirrhosis and HCC [5,7]. HCC develops at about 1-7% per year [5,7]. It has been demonstrated that subjects who achieve sustained virological response (SVR) have a clear advantage at histological and clinical levels compared to those who do not achieve SVR [8-12]. The present standard for the judgment of SVR is undetectability of serum HCV RNA at 24 weeks post-treatment.

Preventive measures against HCV, including vaccine development, are now in progress [13]. But the standard of care (SOC), peginterferon and ribavirin therapy, and new standard of care (NSOC), combination protease inhibitors such as telaprevir or boceprevir with peginterferon plus ribavirin therapy, have been approved for the eradication of HCV in US, Europe, and Japan [14-18]. Even with these advances in antiviral therapies against HCV, SVR rates were ~70% in HCV genotype-1 treated with NSOC and ~80% in HCV genotype-2/3 treated with SOC. Rash also occurs in 56% of patients treated with NSOC, compared to 34% of patients treated with SOC alone. Other adverse events were still present [19], although even interferon is also associated with severe adverse events [20]. When we treat patients infected with HCV in daily clinical practice, it seems important to be aware of the potential treatments of HCV in the near future, as the development of new drugs is always ongoing.

**Figure 1:**
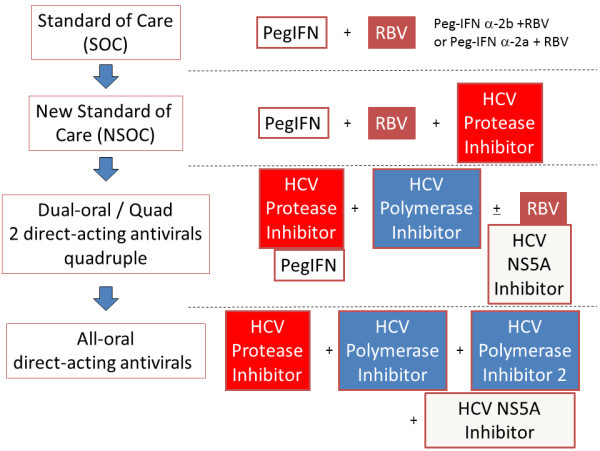
**Treatments for chronic hepatitis C in the present and future.** PegIFN, peginterferon; RBV, ribavirin.

HCV belongs to the flaviviridae family, and HCV genome is a positive-strand ~9.6-kb RNA. HCV has a 5^′^ untranslated region (5^′^UTR), a long open reading frame, and a 3^′^UTR. An internal ribosomal entry site (IRES), containing the 5^′^UTR and part of the core coding region, forms a stem-loop structure and supports translation initiation of HCV genome in a cap-independent manner [21,22]. HCV genome encodes a single precursor polyprotein that is processed by host signal peptidases and HCV proteases, resulting in structural (core, envelopes E1 and E2, and p7) and nonstructural (NS2, NS3, NS4A, NS4B, NS5A and NS5B) proteins. Direct-acting antivirals (DAAs) against HCV are classified into several categories: 1) HCV NS3/4A protease inhibitors, 2) HCV NS5B polymerase inhibitors, 3) HCV NS5A inhibitors, and others. In the near future, interferon-sparing regimens and treatment with all-oral DAAs will play major roles in treating HCV-infected patients (Figure 1).

### Standard of care (SOC) treatment for HCV infection

Interferon, combination interferon plus ribavirin, and peginterferon plus ribavirin increased SVR rate from ~5% to ~40-80%, depending on the HCV genotypes [18,23]. Peginterferon plus ribavirin treatment for 48 weeks, the SOC treatment for HCV genotype 1-infected patients, leads to only ~50% SVR in those patients with high viral loads, who were mostly null-responders or relapsers [23-27]. On the other hand, peginterferon plus ribavirin treatment for 24 weeks, the SOC treatment for HCV genotype 2-infected patients, leads to ~80% SVR in those patients (Table 1). Race has been shown to be a factor in the response to therapy for HCV infection [25,27]. A higher homocysteine level is also one of factors predicting a nonresponse to treatment [28]. Because the favorable interleukin 28B (IL28B) associated-single nucleotide polymorphisms (SNPs), leading to better response, exist at substantially greater frequency in European than African populations, they also explain approximately half of the difference in response rates between African-Americans and patients of European ancestry [29]. IL28B SNPs help to improve the treatment outcomes in HCV-patients treated with SOC [29-34]. In future interferon-included regimens, IL28B SNPs may also provide useful information about the treatment response even before treatment.

**Table 1:** Standard of care treatment and sustained virological response rates for chronic hepatitis C patients

**References**	**G**	**Number of patients**	**Naïve or re-treatment**	**Formula of therapy**	**Duration of treatment (weeks)**	**SVR rates (%)**	**Notes**
[24]	G1	298		Peginterferon alfa-2a plus ribavirin	48	46	
		285		Interferon alfa-2b plus ribavirin	48	36	
		145		Peginterferon alfa-2a plus placebo	48	21	
[24]	G2/G3	140		Peginterferon alfa-2a plus ribavirin	48	76	
		145		Interferon alfa-2b plus ribavirin	48	61	
		69		Peginterferon alfa-2a plus placebo	48	45	
[24]	G4	13		Peginterferon alfa-2a plus ribavirin	48	77	
		11		Interferon alfa-2b plus ribavirin	48	36	
		9		Peginterferon alfa-2a plus placebo	48	22	
[25]	G1 (98%)	100		Peginterferon alfa-2b plus placebo	48	19	Blacks
		100		Peginterferon alfa-2a plus ribavirin	48	52	Non-Hispanic Whites
[26]	G2/3	732		Peginterferon alfa-2a plus ribavirin	16	65	
		731		Peginterferon alfa-2a plus ribavirin	24	76	
[27]	G1	269	naive	Peginterferon alfa-2a plus ribavirin	48	34	Latino
		300	naive	Peginterferon alfa-2a plus ribavirin	48	49	Non-Latino

G, genotype; SVR, sustained virological response.

### New standard of care (NSOC) treatment for HCV infection

In 2011, telaprevir and boceprevir were the first approved DAAs against HCV. The triple combination therapy of telaprevir or boceprevir plus ribavirin and peginterferon-alfa is the NSOC treatment for chronic HCV genotype 1-infected patients [35-42]. Telaprevir (VX-950) is a potent, selective inhibitor of NS3/4A protease, which is essential for HCV replication [43]. SVR rates among treatment-naïve patients were ~70% in telaprevir-included regimens [35,36,38,40]. The SVR rates among patients with no previous response were 30~40% and those among patients with a previous relapse were 70~75%, both in telaprevir-included regimens [37,39]. SVR rates were significantly higher in telaprevir-included regimens than in SOC among patients who had a previous relapse (83-88% vs. 24%), a partial response (54-59% vs. 15%) and non- response (29-33% vs. 5%) [39,44]. Thus, SVR rates were higher among patients who had previously had relapses than among nonresponders. Response-guided therapy is also useful in the telaprevir-included regimen (Table 2) [40]. Boceprevir (SCH 503034) is a potent ketoamide inhibitor of HCV NS3 serine protease [45]. The addition of boceprevir to SOC results in higher SVR rates in both treatment-naïve and re-treated patients infected with HCV genotype 1 (Table 3) [41,42]. SVR rates were significantly higher in boceprevir-included regimens than in SOC among patients who had a prior relapse (69-75% vs. 29%) or a prior nonresponse (40-52% vs. 7%) [42]. Viewed from the clinical data of first-generation protease inhibitors, telaprevir and boceprevir, these drugs showed potent inhibition of HCV, although they occasionally led to severe adverse events [46,47].

**Table 2:** New standard of care treatment with telaprevir for chronic hepatitis C patients

**References**	**G**	**Number of patients**	**Naïve or re-treatment**	**Formula of therapy, and duration of treatment (weeks)**	**SVR rates (%)**	**Notes**
[35]	G1	79	naive	T12PR24	61	PROVE1 Study
		79	naive	T12PR48	67	
		17	naive	T12PR12	35	
		75	naive	PR48	41	
[36]	G1	81	naive	T12PR24	69	PROVE2 Study
		82	naive	T12PR12	60	
		78	naive	T12P12	36	
		42	naive	PR48	46	
[37]	G1	115	re-treatment	T12PR24	51	PROVE3 Study
		113	re-treatment	T12PR48	53	
		111	re-treatment	T24P24	24	
		114	re-treatment	PR48	14	
[38]	G1	363	naive	T12PR	73	ADVANCE Study
		364	naive	T8PR	67	
		49	naïve	PR48	44	
[39]	G1	145	re-treatment	T12PR48	93	REALIZE Study
		141	re-treatment	Lead-in T12 PR48	89	
		68	re-treatment	PR48	24	
[40]	G1	162	naïve	T12PR24 (RGT)	92	ILLUMINATE Study
		160	naïve	T12PR48 (RGT)	88	
		118	naïve	T12PR48 (non-RGT)	64	
		100	naïve	Discontinued treatment before wk 20	23	

**Table 3:** New standard of care treatment with boceprevir for chronic hepatitis C patients

**References**	**G**	**Number of patients**	**Naïve or re-treatment**	**Formula of therapy, and duration of treatment (weeks)**	**SVR rates (%)**	**Notes**
[41]	G1	363	naive	PR48	38	SPRINT-2
		368	naive	PR4+BocPR24+PR22	63	
		366	naive	PR4+BocPR44	66	
[42]	G1	80	re-treatment	PR48	21	HCV RESPOND-2
		162	re-treatment	PR4+BocPR24+PR22	59	
		161	re-treatment	PR4+BocPR44	66	

PR48 group received peginterferon alfa-2a and ribavirin for 48 weeks; T12PR12, T12PR24 or T12 PR48 group received telaprevir for 12 weeks and peginterferon alfa-2a and ribavirin for 12, 24 or 48 weeks, respectively; T12P12 group received telaprevir and ribavirin for 12 weeks; T24P24 group received telaprevir and peginterferon alfa-2a for 24 weeks; RGT, response-guided therapy; Boc, boceprevir.

### Second-generation HCV NS3/4A inhibitors

Simeprevir (TMC435) is an investigational HCV NS3/4A protease inhibitor administered orally once daily, and it is currently in phase III clinical development [48]. It differs from the first generation protease inhibitors in terms of its once-daily administration. Superior efficacies of simeprevir and peginterferon plus ribavirin were observed compared to those of peginterferon plus ribavirin alone in treatment-naive [49] and previously treated patients (Table 4) [50]. Although anemia and rash, respectively, were notable adverse events in boceprevir and telaprevir, those of simeprevir and peginterferon plus ribavirin did not differ from those of peginterferon plus ribavirin alone in phase II studies [48-50].

**Table 4:** New standard of care treatment with other drugs (second-generation DAAs) chronic hepatitis C patients

**References**	**G**	**Number of patients**	**Naïve or re-treatment**	**Formula of therapy, and duration of treatment (weeks)**	**SVR rates (%)**	**Notes**
[48,49]	G1	78	Naïve	TMC435 12 W peginterferon/ribavirin RGT	82.1	TMC435 (simeprevir) 75 mg daily
		75		TMC435 24 W peginterferon/ribavirin RGT	74.7	
		77		TMC435 12 W peginterferon/ribavirin RGT	80.5	TMC435 150 mg daily
		79		TMC435 24 W peginterferon/ribavirin RGT	86.1	
		77		Placebo	64.9	
[48,50]	G1	27	Relapsers	PR PR48	37	
		79		TMC435 100 mg PR48	85	
		79		TMC435 150 mg PR48	85	
		23	Partial responders	PR PR48	9	
		68		TMC435 100 mg PR48	57	
		69		TMC435 150 mg PR48	75	
		16	Null responders	PR PR48	19	
		50		TMC435 100 mg PR48	46	
		51		TMC435 150 mg PR48	51	
[55]	G1	12		3 mg daclatasvir PR48	42	Treatment-naïve or less than 4 wks of exposure to ribavirin or interferon-based therapy
		12		10 mg daclatasvir PR48	83	
		12		60 mg daclatasvir PR48	83	
		12		Placebo PR48	25	

MK-5172, a novel P2-P4 quinoxaline macrocyclic peptide, maintained potency across a genetically diverse panel of genotype 1a and 1b sequences from plasma of HCV-infected patients. This drug is to be used in combination with peginterferon plus ribavirin or with other DAAs [51]. Faldaprevir (BI 201335) is an inhibitor of HCV NS3/4A protease and is undergoing phase III clinical trials [52,53].

### HCV NS5A and NS5B inhibitors

HCV NS5A inhibitor daclatasvir (BMS-790052) with potent clinical effects has been found in the HCV replicon system [54]. Daclatasvir is a potent NS5A replication complex inhibitor and increases the antiviral potency of peginterferon and ribavirin [55] (Tables 4).

**Table 5:** New drugs (DAA combinations) for the treatment of hepatitis C in phase II study

**Drug name/category**	**Drug name/category**	**Company**
ABT-450r/Protease inhibitor	ABT-072/Polymerase inhibitor	Abbott/Enanta
ABT-450r/Protease inhibitor	ABT-267/NS5A inhibitor	Abbott/Enanta
ABT-450r/Protease inhibitor	ABT-333/Polymerase inhibitor	Abbott/Enanta
Daclatasvir (BMS-79002)/ NS5A inhibitor	Sofosbuvir (GS-7977)/ Polymerase inhibitor	Bristol-Myers Squibb/Gilead
Daclatasvir (BMS-79002)/ NS5A inhibitor	Simeprevir (TMC435)/ Protease inhibitor	Bristol-Myers Squibb/Janssen
Danoprevir (RG7227)/ Protease inhibitor	Setrobuvir (ANA 598)/ Polymerase inhibitor	Genentech
Danoprevir (RG7227)/ Protease inhibitor	Mericitabine (RG7128)/ Polymerase inhibitor	Genentech
GS-9256	Tegobuvir (GS-9190)/ Polymerase inhibitor	Gilead
Incivek (Telaprevir)/ Protease inhibitor	VX-222/Polymerase inhibitor	Vertex
Mericitabine (RG7128)/ Polymerase inhibitor	Incivek (Telaprevir)/ Protease inhibitor	Genentech/Vertex
Simeprevir (TMC435)/ Protease inhibitor	Daclatasvir (BMS-79002)/ NS5A inhibitor	Janssen/Bristol-Myers Squibb
Simeprevir (TMC435)/ Protease inhibitor	Sofosbuvir (GS-7977)/ Polymerase inhibitor	Janssen/ Gilead
Simeprevir (TMC435)/ Protease inhibitor	TMC647055/NNI inhibitor/Ritonavir	Janssen
Sofosbuvir (GS-7977)/ Polymerase inhibitor	GS-5885/ NS5A inhibitor	Gilead
VX-135/Polymerase inhibitor	GSK2336805/ NS5A inhibitor	Vertex/GSK
VX-135/Polymerase inhibitor	Simeprevir (TMC435)/ Protease inhibitor	Vertex/Janssen

HCV advocate: http://hcvadvocate.blogspot.ca/p/quick-reference-guide_21.html accessed on 2013/2/24.

**Table 6:** New drugs for the treatment of hepatitis C in phase III study (combinations with peginterferon plus ribavirin, or DAA combinations)

***Combination with peginterferon plus ribavirin***		
**Drug name**	**Category**	**Company**
Daclatasvir (BMS-79002)	NS5A inhibitor	Bristol-Myers Squibb
Simeprevir (TMC435)	Protease inhibitor	Janssen
Sofosbuvir (GS-7977)	Polymerase inhibitor	Gilead
***DAA combinations***		
**Drug name/Category**	**Drug name/Category**	**Company**
ABT-450r/Protease inhibitor	ABT-267/NS5A inhibitor and/or ABT-333/Polymerase inhibitor	Abbott/Enanta
Daclatasvir (BMS-79002)/ NS5A inhibitor	Asunaprevir (BMS-650032)/Protease inhibitor	Bristol-Myers Squibb
Faldaprevir (BI201335)/ Protease inhibitor	GS-5885/NS5A inhibitor	Boehringer Ingelheim
Sofosbuvir (GS-7977)/ Polymerase inhibitor	BI207127/Polymerase inhibitor	Gilead
Sofosbuvir (GS-7977)/ Polymerase inhibitor	GS-5885/NS5A inhibitor	Gilead

HCV advocate: http://hcvadvocate.blogspot.ca/p/quick-reference-guide_21.html accessed on 2013/2/24.

Sofosbuvir (GS-7977/PSI-7977) is a nucleotide inhibitor of HCV NS5B polymerase. Triple therapy including peginterferon plus ribavirin and sofosbuvir cures >90% of patients treated for 12 or 24 weeks regardless of HCV genotype [56]. However, SVR rates of sofosbuvir plus ribavirin were ~76% for interferon-naive patients and ~11% for prior null responders in HCV genotype 1 patients [56,57]. Sofosbuvir plus peginterferon/ribavirin in treatment-naive patients with HCV genotype 1 leads to higher RVR rates and SVR rates than SOC (88-94% vs. 21%; and 56-83% vs. 43%, respectively) [58]. It was also reported that sofosbuvir in combination with low- or full- dose ribavirin for 24 weeks leads to higher efficacy in difficult-to-treat HCV infected genotype 1 patients [59].

### HCV drugs in the future

New drugs for the treatment of hepatitis C are now being investigated in phase II and phase III clinical trials (Tables 5 and 6). These include interferon-sparing regimens, which are needed for the treatment of those intolerant to, or medically ineligible for peginterferon plus ribavirin therapy [60,61]. Among treatment-experienced patients with advanced fibrosis or cirrhosis, previous relapsers are likely to respond very well to telaprevir- or boceprevir-based treatment, although advanced liver disease had a greater influence on SVR rates in previous nonresponders [39,42,62,63]. Of importance is interferon-sparing combinations that might potentially be used in all patients who cannot use interferon such as subjects with decompensated cirrhosis or low platelet count. Some DAAs are potent inhibitors independently of HCV genotypes [56,64-66]. The all-oral combination of daclatasvir plus sofosbuvir, with or without ribavirin, leads to higher SVR rates in treatment-naive patients chronically infected with HCV genotypes 1, 2 and 3 [67]. We may select the combination of several drugs according to personal features and/or HCV genotypes. In the near future, all-oral DAAs will treat HCV-infected patients (Figure 1).

## Conclusions

We expect that all-oral DAAs and interferon-free regimens will be applied in the treatment of HCV-infected patients and that they will have more potent efficacy and less adverse events. Further studies are now ongoing.

## Competing interests

Dr. Tatsuo Kanda reports receiving lecture fees from Chugai Pharmaceutical, MSD, Ajinomoto, GlaxoSmithKlein and Mitsubishi Tanabe Pharma, and Prof. Osamu Yokosuka received grant support from Chugai Pharmaceutical, Bayer, MSD, Daiichi-Sankyo, and Mitsubishi Tanabe Pharma.

## Authors’ contributions

TK drafted the manuscript and all authors read through and made corrections to the manuscript. All authors read and approved the final manuscript.
